# MEETINGS CALENDAR - 2016

**Published:** 2016

## November

**10 to 11 - BISMICS (British and Irish Society for Minimally Invasive Cardiac Surgery)**
Birmingham, United Kingdom
Informations: Lorraine Richardson
Phone: +447711132946
Fax: 01296 733823
E-mail: lorrainerichardson1@btinternet.com
Site: www.bismics.org.uk

**11 to 13 - AATS International Coronary Congress**
New Delhi, India
Informations: Dr. Kunal Sarkar
Phone: +919830080006
E-mial: kunal.cardiac@gmail.com
Site: www.icc2016.in

**11 - Horizons: Thoracic Intervention and Surgery to Cure Lung Cancer**
London, United Kingdom
Informations: Alison Chan
Phone: 02072903937
Fax: 02072903992
E-mail: cardiothoracic@rsm.ac.uk
Site: www.rsm.ac.uk/events/cth01

**14 to 18 - ESTS Course on TB and other lung diseases of Surgical Interest**
Tradate, Italy
Informations: Sue Hesford
Phone: + 44 1392 430671
Fax: + 44 1392 430671
E-mail: sue@ests.org.uk
Site: www.ests.org

**15 to 18 - EACTS Academy: Congenital Heart Disease**
Windsor, United Kingdom
Informations: EACTS
Site: http://www.eacts.org/academy/2016-programme/

**17 and 18 - Controversies and Advances in the Treatment of Cardiovascular Disease: The Sixteenth in the Series**
Beverly, United States
Informations: Rebecca Law
Phone: 760-720-2263
Fax: 760-720-6263
E-mail: rlaw@promedicacme.com
Site: http://promedicacme.com/

**24 - Electromagnetic Navigation Bronchoscopy Workshop**
Hong Kong S.A.R., China
Informations: Division of Cardiothoracic Surgery, Department of Surgery The Chinese University of Hong Kong
Phone: (852) 2632 2629/2632 3586
Fax: (852) 2637 7974
E-mail: enb2016@surgery.cuhk.edu.hk
Site: www.surgery.cuhk.edu.hk/enb2016

**24 and 25 - ESTS International Leadership and Management (ILM) Development Program**
Leeds, United Kingdom
Informations: Sue Hesford
E-mail: sue@ests.org.uk
Site: www.ests.org

**24 and 25 - EACTS Academy: Modern Perspectives on Atrial Fibrillation Surgery**
Windsor, United Kingdom
Informations: EACTS
Site: http://www.eacts.org/academy/2016-programme/

**28 and 29 - EACTS Academy: Hospital Leadership**
Windsor, United Kingdom
Informations: EACTS
Site: http://www.eacts.org/academy/2016-programme/

## December

**1 - 5^th^ LECCE VATS Lobectomy Course**
Lecce, Italy
Informations: Gaetano Di Rienzo
Phone: +39/3496398344
Fax: +39/0832661608
E-mail: gaetanodirienzo1@gmail.com
Site: chirurgiatoracicalecce.it

**4 to 6 - ICI Meeting 2016**
Tel Aviv, Israel
Informations: Paragon Israel (Dan Knassim)
Phone: +972-3-5767730/7
E-mail: secretariat@icimeeting.com
http://2016.icimeeting.com/

**5 and 6 - 13^th^ European Cardiology Congress**
Madrid, Spain
Informations: David Mathews
Phone: 7025085200
E-mail: eurocardiology@cardiologyconference.org
Site: http://cardiology.conferenceseries.com/europe/

